# Agreement and classification performance of the heaviness of smoking index (HSI) compared with the Fagerström test for cigarette dependence (FTCD): A subgroup analysis

**DOI:** 10.1016/j.abrep.2026.100740

**Published:** 2026-08-25

**Authors:** Abdurrahman Charkazi, Seyed Ghadir Hosseini, Hajar Dehghan, Arezou Foroughi, Bagher Pahlavanzadeh

**Affiliations:** a Associate Professor of Health Education & Promotion, Environmental Health Research Center, Faculty of Health, Golestan University of Medical Sciences, Gorgan, Iran; b Assistant Professor of Health Education and Promotion, Department of Public Health, Faculty of Health, Golestan University of Medical Sciences, Gorgan, Iran; c Department of Nursing, Gonbad Kavous School of Nursing, Golestan University of Medical Sciences, Gonbad Kavous, Iran; d Health Care, Education Organization, Gonbad Kavous County, Gonbad Kavous, Iran; e Assistant Professor of Biostatistics, Research Center for Environmental Contaminants (RCEC), Abadan University of Medical Sciences, Abadan, Iran

**Keywords:** HSI, FTCD, Nicotine addiction, Cigarette smoking, ROC analysis

## Abstract

**Background:**

The Heaviness of Smoking Index (HSI) is a brief tool for assessing nicotine addiction. This study evaluated the agreement and classification performance of the HSI relative to the Fagerström Test for Cigarette Dependence (FTCD).

**Methods:**

In this cross-sectional study of 1147 adult smokers, HSI scores were derived from the FTCD. Agreement and apparent classification performance were examined using Pearson correlation, intraclass correlation coefficient (ICC), Cohen's kappa, receiver operating characteristic (ROC) analysis, and Bland–Altman plots across demographic and behavioral subgroups.

**Results:**

HSI showed a strong correlation with FTCD (*r* = 0.904) and excellent continuous agreement (ICC = 0.95). Categorical agreement for high nicotine addiction was substantial (κ ≈ 0.65). ROC analysis demonstrated excellent apparent classification performance (AUC = 0.942) for identifying FTCD-defined severe addiction. An HSI cut-off of ≥3 (derived from an optimal threshold of 2.5) provided a favorable balance between sensitivity and specificity for initial screening, whereas a cut-off of ≥4 showed high specificity for severe addiction. Performance remained generally consistent across subgroups, although some subgroup-specific variation was observed. Bland–Altman analysis indicated minimal mean bias, although HSI proportionally underestimated addiction at higher severity levels.

**Conclusion:**

HSI demonstrates strong agreement with FTCD and may serve as a rapid screening measure for assessing nicotine addiction. A lower cut-off (≥3) may be useful for initial screening, whereas a higher cut-off (≥4) may help identify severe addiction. Caution is warranted at higher addiction levels and among poly-tobacco users.

## Highlights

• HSI showed strong correlation and excellent agreement with the Fagerström Test for Cigarette Dependence (FTCD).
• HSI showed high apparent classification performance for identifying FTCD-defined severe nicotine addiction.
• HSI cut-off ≥3 balanced sensitivity and specificity for screening; ≥4 showed high specificity for severe addiction.
• Agreement and classification performance of the HSI were consistent across demographic and behavioral subgroups.
• HSI underestimated addiction severity at higher levels, warranting cautious interpretation in highly addicted smokers.

## 1 Introduction

Tobacco use remains one of the leading causes of preventable morbidity and mortality worldwide. The Global Burden of Disease Study 2020 estimated that tobacco use is responsible for nearly eight million deaths annually, reflecting its substantial health burden despite ongoing global tobacco control efforts (GBD 2019 Tobacco Collaborators., 2021). Chronic exposure to tobacco smoke is associated with a wide range of adverse health outcomes, including cardiovascular disease, chronic obstructive pulmonary disease, and multiple malignancies (U.S. Department of Health and Human Services, 2020; National Cancer Institute, 2010).

In Iran, findings from the 2021 WHO STEPwise approach to Surveillance (STEPS) survey indicate that approximately 14.0% of individuals aged 15 years and older (including adolescents and adults) currently use tobacco, with marked sex differences in prevalence (25.9% among men and 4.4% among women) (Abbasi-Kangevari et al., 2023). Previous epidemiological evidence indicates that smoking prevalence and nicotine addiction vary across age groups, with younger smokers generally exhibiting lower cumulative exposure but higher quit intentions, whereas older smokers tend to have longer smoking histories and greater levels of addiction (Abbasi-Kangevari et al., 2023). Beyond these demographic differences, smoking behaviors and nicotine addiction have been shown to vary according to smoking intensity and readiness to quit, underscoring the need for valid and efficient measures of nicotine addiction that perform consistently across diverse smoker subgroups(Pérez-Ríos et al., 2009; Prochaska & DiClemente, 1983). This high public health burden underscores the urgent need for brief, reliable, and easily administered screening tools in clinical settings to identify high- addiction smokers and facilitate targeted cessation interventions (Fiore et al., 2008; World Health Organization, 2019).

Nicotine exposure through cigarette smoking leads to the development of nicotine addiction, a central neurobiological and behavioral mechanism underlying the maintenance of smoking behavior and resistance to cessation (Benowitz et al., 1988; Benowitz & Henningfield, 1994). Nicotine addiction encompasses both physiological and behavioral components, including tolerance, withdrawal symptoms, and habitual patterns of use that reinforce continued smoking (American Psychiatric Association, 2022; U.S. Department of Health and Human Services, 1989). As smoking prevalence declines in some populations, remaining smokers tend to exhibit higher levels of addiction, a pattern consistent with the “hardening hypothesis,” which suggests that individuals least likely to quit become increasingly concentrated among continuing smokers (Darville & Hahn, 2014; Flor et al., 2021). This shift highlights the importance of accurate, efficient, and scalable tools for assessing nicotine addiction in both clinical and epidemiological contexts.

Several instruments have been developed to assess nicotine addiction. These include the six-item Fagerström Test for Nicotine Dependence (FTND; Heatherton et al., 1991), the 68-item Wisconsin Inventory of Smoking Dependence Motives (Piper et al., 2004), the 19-item Nicotine Dependence Syndrome Scale (Shiffman et al., 2004), the 11-item Glover–Nilsson Smoking Behavioral Questionnaire (Glover et al., 2005), the 12-item Cigarette Dependence Scale (Etter, 2005), and the 10-item Hooked on Nicotine Checklist (Wellman et al., 2005). The FTND was subsequently renamed the Fagerström Test for Cigarette Dependence (FTCD) to better reflect that it specifically assesses cigarette dependence rather than nicotine addiction across different tobacco or nicotine products (Fagerström, 2012). The FTCD remains one of the most widely used instruments for assessing nicotine addiction among cigarette smokers and has been extensively utilized in both research and clinical settings.

Although the FTCD is one of the most widely used instruments for assessing nicotine addiction in cigarette smokers and has well-established psychometric properties, its six-item format may be less practical in settings where rapid assessment is required. Brief instruments may be advantageous in large-scale epidemiological surveys, primary care settings, and smoking cessation clinics, where efficient assessment is important (Fiore & Baker, 2011; Rolstad et al., 2011). In such contexts, shorter measures derived from the FTCD may provide a practical approach for rapid screening, provided that their performance relative to the full FTCD is appropriately evaluated (Fiore & Baker, 2011; Rolstad et al., 2011).

In Iran, where national epidemiological surveys such as the WHO STEPS survey and routine primary care settings where consultations are often time-limited require efficient assessment approaches, evaluating the agreement and classification performance of the two-item HSI relative to the FTCD is particularly relevant. However, the only previous Iranian study examining HSI–FTCD agreement was limited to a relatively small sample of male smokers and did not evaluate performance across important demographic and behavioral subgroups. Therefore, assessing the HSI in a larger and more diverse sample of Iranian smokers may provide more generalizable evidence regarding its potential utility in clinical practice and epidemiological research.

The Heaviness of Smoking Index (HSI), derived from two core FTCD items—time to first cigarette after waking and cigarettes smoked per day—was introduced as a brief indicator of nicotine addiction (Heatherton et al., 1989; Heatherton et al., 1991).

Psychometric studies have shown that these two items represent important components of the nicotine addiction dimension assessed by the FTCD. With a score range of 0–6, the HSI provides a rapid assessment of smoking intensity-related addiction severity and has been widely used in population-based surveys, primary care settings, brief interventions, and smoking cessation programs (Kozlowski et al., 1994). Previous studies have demonstrated associations between HSI scores and longer measures of nicotine addiction, supporting its practical utility in settings where rapid assessment is required (Borland et al., 2010; Chaiton et al., 2007; Diaz et al., 2005).

Previous studies have demonstrated moderate to strong correlations and acceptable levels of agreement between the HSI and the full FTCD (Borland et al., 2010; Diaz et al., 2005; Pérez-Ríos et al., 2009); however, evidence regarding its classification agreement, optimal cut-off points, and agreement varies across populations and study designs (Chabrol et al., 2005; Lim et al., 2022; Pérez-Ríos et al., 2009). Moreover, relatively few studies have applied comprehensive agreement-based approaches, including receiver operating characteristic (ROC) analysis and subgroup evaluations, to fully assess its performance.

Despite their widespread use, both FTCD and HSI have important limitations. Because the HSI is directly derived from two FTCD items, estimates of agreement and classification performance between the two measures are subject to criterion contamination (incorporation bias), which may inflate correlation, agreement, and classification agreement estimates due to shared item content rather than independent construct validity. In addition, both instruments were originally developed for cigarette smoking and may not fully capture nicotine addiction in individuals using multiple tobacco products, an increasingly important issue in countries such as Iran where concurrent use of cigarettes, hookah, nass, and electronic cigarettes is common(Abbasi-Kangevari et al., 2023). Therefore, the present study is framed primarily as an agreement and comparative performance analysis rather than an independent validation study.

In Iran, only one previous study has examined agreement between the HSI and FTCD. In a sample of 230 male smokers, Pahlavanzadeh and Charkazi reported substantial agreement between the two instruments and suggested that the HSI may serve as a practical alternative for rapid assessment of nicotine addiction (Pahlavanzadeh & Charkazi, 2024). However, that study was restricted to men, included a considerably smaller sample, and did not evaluate agreement across demographic and behavioral subgroups or assess classification agreement using ROC-based methods. Consequently, the generalizability of those findings to women and other clinically relevant smoking subgroups remains uncertain.

Moreover, little is known about whether the performance of HSI remains stable across demographic and behavioral subgroups, particularly those defined by smoking intensity and readiness to quit smoking. According to the Transtheoretical Model (TTM), readiness to quit smoking can be categorized into five stages: precontemplation, contemplation, preparation, action, and maintenance (Prochaska & DiClemente, 1983). Because nicotine addiction and cessation motivation vary across these stages, evaluating the stability of measurement performance across TTM stages is essential for ensuring robustness of screening tools across behavioral contexts.

Addressing these gaps is important given the potential utility of the HSI as a rapid screening tool in clinical practice, primary care, population-based surveys, and smoking cessation programs. The present study extends previous Iranian evidence by including both men and women, recruiting a substantially larger sample, evaluating agreement using multiple complementary approaches (correlation, ICC, kappa, and Bland–Altman analysis), assessing apparent classification agreement through ROC analysis, and examining the stability of findings across demographic and behavioral subgroups.

Given the time and resource constraints within the Iranian healthcare system, evaluating whether the HSI can serve as a rapid and reliable screening tool has practical importance. Accordingly, the present study aimed to evaluate the agreement between the HSI and the Fagerström Test for Cigarette Dependence (FTCD) in a large and diverse sample of Iranian smokers. Specifically, we assessed agreement between the two measures, examined classification agreement using ROC-based methods, explored performance across demographic and behavioral subgroups, and identified optimal cut-off points. Based on previous evidence reporting moderate to strong correlations between the HSI and FTCD (Borland et al., 2010; Pérez-Ríos et al., 2009), and the objectives of the present study, the following hypotheses were formulated:

1. HSI demonstrates a strong positive correlation and high agreement with the FTCD, as measured by the Pearson correlation coefficient and intraclass correlation coefficient (ICC).
2. There is substantial agreement between the HSI and FTCD in classifying smokers according to levels of nicotine addiction, as assessed by Cohen's kappa statistic.
3. The HSI demonstrates good apparent classification performance for identifying FTCD-defined high nicotine addiction, as evaluated using receiver operating characteristic (ROC) analysis.
4. The agreement and apparent classification performance of the HSI relative to the FTCD remain generally consistent across demographic and behavioral subgroups, while allowing for potential subgroup-specific variation.

## 2 Methods

### 2.1 Participants

A total of 1147 individuals participated in the study, with a mean age of 34.6 years (SD: 11.3), ranging from 18 to 70 years, the majority of whom were male (74.8%). The mean age of smoking initiation was 20.3 years (SD: 6.5), and the average number of cigarettes smoked per day was 13.9 (SD: 9.7). Regarding consumption patterns, 45.7% of participants used other tobacco products in addition to cigarettes, including hookah, nass, vaping, or pipes; 47.9% of the total sample were classified as dual users, and 2.7% as poly-tobacco users. Based on cumulative smoking exposure (pack-year index), the majority of participants (80.8%) were classified as light smokers. The prevalence of high nicotine addiction was 36.4% according to the FTCD (score ≥ 6) and 27.1% according to the HSI (score ≥ 4) (Table 1).

**Table 1:** Demographic and smoking-related characteristics of the study participants (n = 1147).

Variable	Category	*n*	%
Gender	Female	289	25.20
	Male	858	74.80
Age group (years)	<30	459	40.02
	30–39	324	28.25
	40–49	245	21.36
	50–59	91	7.93
	≥60	28	2.44
Smoking Initiation Age (years)	<20	547	48.28
	≥20	586	51.72
Any Other Tobacco Use	No	623	54.32
	Yes	524	45.68
Poly-tobacco Status	Exclusive cigarette user	567	49.43
	Dual user	549	47.87
	Poly-tobacco user	31	2.70
Stage of Change	Precontemplation	620	54.05
	Contemplation	271	23.63
	Preparation	180	15.69
	Action	21	1.83
	Maintenance	55	4.79
Cumulative Smoking Exposure (pack-year)	Light smoker	902	80.75
	Moderate smoker	174	15.58
	Heavy smoker	41	3.67
Addiction Level (FTCD)	Low (<6)	730	63.64
	High (≥6)	417	36.36
Addiction Level (HSI)	Low (<4)	836	72.89
	High (4–6)	311	27.11

*Note.* FTCD = Fagerström Test for Cigarette Dependence; HSI = Heaviness of Smoking Index. Percentages may not total exactly 100 because of rounding.

For subgroup analyses, participants were categorized according to demographic and behavioral characteristics, including age group, gender, cumulative smoking exposure, stage of change, any other tobacco use, and poly-tobacco status.

Cumulative smoking exposure categories were classified according to pack-year index values as light (<20 pack-years), moderate (20–38 pack-years), and heavy (>38 pack-years). Rather than using fixed clinical guidelines, these specific cut-off points were empirically derived from the percentile distribution of the current sample to ensure approximately balanced group sizes and sufficient statistical power for the subsequent subgroup analyses. “Dual users” referred to participants who used cigarettes plus one additional tobacco/nicotine product, whereas “poly-tobacco users” referred to those using cigarettes plus two or more additional tobacco/nicotine products (e.g., hookah, nass, or vaping devices). Stages of change were classified according to the Transtheoretical Model of behavior change, including precontemplation (no intent to quit), contemplation (considering quitting within 6 months), preparation (planning to quit within 30 days), action (recent quit attempt), and maintenance (sustained abstinence >6 months) stages.

### 2.2 Procedure

Data were collected through face-to-face administration of the questionnaires. Trained research assistants were present while participants completed the questionnaires and were available to answer questions or provide clarification when needed. Participants were classified as current cigarette smokers if they had smoked at least 100 cigarettes in their lifetime and had smoked at least one cigarette during the past 30 days. For participants in the action and maintenance stages of smoking cessation, the FTCD and HSI were completed retrospectively based on their smoking behavior during the period when they were actively smoking, using self-reported smoking history obtained from the demographic questionnaire and the FTCD.

Eligibility criteria required participants to have smoked at least 100 cigarettes during their lifetime. (Centers for Disease Control and Prevention (CDC), 2023). Convenience community sampling was employed, whereby participants were recruited from public gathering areas in the cities of the province, such as parks, addiction treatment centers, and cafés. A total of 1147 participants were enrolled, including current smokers and individuals classified in the action (*n* = 21) and maintenance (*n* = 55) stages of behavioral change.

Data were collected between April and December 2025. Participants were assured that their information would remain confidential and that providing their names was not required. The study protocol was approved by the Institutional Review Board (IRB) of Golestan University of Medical Sciences, Vice-Chancellery for Research and Technology (IR.GOUMS.REC.1494.084). The study was conducted in accordance with the Declaration of Helsinki and its later amendments or comparable ethical standards.

### 2.3 Measures

Data were collected using the following questionnaires:

1. Demographic Questionnaire: This questionnaire included information on age, marital status, occupation, education, monthly income, number of cigarettes smoked per day, age of smoking initiation, years of smoking, and use of other forms of tobacco such as hookah, electronic cigarettes, and nass.
2. Fagerström Test for Cigarette Dependence (FTCD): The FTCD is a six-item self-report questionnaire and one of the most widely used tools for assessing nicotine addiction. It evaluates physiological indicators of addiction and has been extensively utilized in research. The FTCD assesses the number of cigarettes smoked per day (fewer than 10, 11–20, 21–30, or more than 30, scored 0–3, respectively), the time to the first cigarette after waking (scored 3 if smoked within 5 min, 2 for 6–30 min, 1 for 31–60 min, and 0 for more than 60 min), smoking behavior when ill (Yes = 1, No = 0), smoking in prohibited places (Yes = 1, No = 0), difficulty refraining from smoking in the morning or at other times (Yes = 1, No = 0), and enjoyment of the first cigarette in the morning or subsequent cigarettes (Yes = 1, No = 0). The total score ranges from 0 to 10, with higher scores indicating greater nicotine addiction (Heatherton et al., 1991). The validity and reliability of this questionnaire have been confirmed in Iran (Sarbandi et al., 2015).
3. Heaviness of Smoking Index (HSI): The HSI is a brief measure derived from two items of the Fagerström Test for Cigarette Dependence: cigarettes smoked per day (scored 0–3) and time to first cigarette after waking (scored 0–3). The index was originally proposed by Heatherton et al. (Heatherton et al., 1989; Heatherton et al., 1991). Scoring details are as per FTCD items. The total score ranges from 0 to 6, with higher scores indicating greater nicotine addiction. Nicotine addiction was classified as high if the FTCD score was ≥6 and the HSI score was ≥4, consistent with widely accepted cut-off points used in previous studies (Chabrol et al., 2005; Heatherton et al., 1991).

### 2.4 Analysis

Continuous variables were described using means ± standard deviations, and categorical variables were presented as frequencies and percentages. Pearson's correlation coefficient was used to examine the linear relationship between FTCD and HSI scores as a descriptive indicator of convergent validity between the two measures of nicotine addiction. Given the difference in the score ranges of the two instruments (FTCD: 0–10; HSI: 0–6), for analyses directly assessing numerical agreement, scores were linearly rescaled to a 0–1 range prior to agreement analyses.

To test the four predefined study hypotheses presented at the end of the Introduction, the following analyses were conducted: Pearson correlation and intraclass correlation coefficient (ICC) for Hypothesis 1, Cohen's kappa statistic for Hypothesis 2, receiver operating characteristic (ROC) analysis for Hypothesis 3, and exploratory subgroup analyses for Hypothesis 4.

A two-way random-effects intraclass correlation coefficient (ICC) was then used to evaluate agreement and reproducibility. The magnitude of ICC values was interpreted according to Cicchetti's criteria, whereby values <0.40 indicate poor, 0.40–0.59 fair, 0.60–0.74 good, and ≥ 0.75 excellent agreement (Cicchetti, 1994). Additionally, Bland–Altman plots were employed to identify systematic bias.

To assess agreement in categorical classifications of addiction level (high/low), simple Cohen's Kappa was calculated. The classification agreement ability of the HSI to identify high nicotine addiction (based on the commonly used cutoff of FTCD ≥6) was evaluated using receiver operating characteristic (ROC) curve analysis, and the optimal cutoff point was determined via Youden's index. ROC analysis assessed the apparent classification performance of the HSI using the FTCD high-addiction classification (≥6) as the reference standard. Because the HSI is derived from two FTCD items (cigarettes per day and time to first cigarette), these measures are not statistically independent; therefore, ROC findings should be interpreted as indicators of apparent classification performance rather than independent classification agreement.

To examine the stability of HSI–FTCD classification agreement across different dimensions of smoking exposure, subgroup analyses were performed based on smoking duration, current smoking intensity, and cumulative smoking exposure. Smoking duration (years smoked) was categorized into tertiles based on the sample distribution: low (0–7 years), medium (8–15 years), and high (≥16 years). Current smoking intensity (daily cigarette consumption) was similarly divided into tertiles: low (0–8 cigarettes/day), medium (9–20 cigarettes/day), and high (≥21 cigarettes/day). These tertile-based classifications yielded approximately equal-sized subgroups (approximately 382–383 participants per group). Cumulative smoking exposure (pack-years) was categorized into light (<20 pack-years), moderate (20–38 pack-years), and heavy (>38 pack-years), with cut-offs derived from the sample distribution to ensure balanced groups.

Additional subgroup analyses were conducted across demographic and behavioral strata, including gender, age group, stage of change, and tobacco-use patterns (any other tobacco use and poly-tobacco status) as presented in Table 1. Subgroup analyses were exploratory and descriptive in nature and were intended to evaluate the stability of agreement and classification performance across different participant characteristics. No formal statistical comparisons were performed between subgroups. All agreement and classification performance measures were reported with 95% confidence intervals. Analyses were performed using R software version 4.4.2, with statistical significance set at *p* < 0.05.

## 3 Results

### 3.1 Agreement, and categorical agreement

Correlation analysis indicated a strong positive relationship between the FTCD and HSI (*r* = 0.904, 95% CI: 0.893–0.914, *P* < 0.001). After linear rescaling of both scores to a 0–1 range, the intraclass correlation coefficient demonstrated excellent agreement (ICC = 0.950, 95% CI: 0.944–0.956). Additionally, Cohen's kappa showed substantial agreement between the two instruments in classifying smokers into high nicotine addiction versus other categories (κ = 0.65, 95% CI: 0.60–0.69).

### 3.2 Systematic bias (bland–altman analysis)

**Fig. 1:**
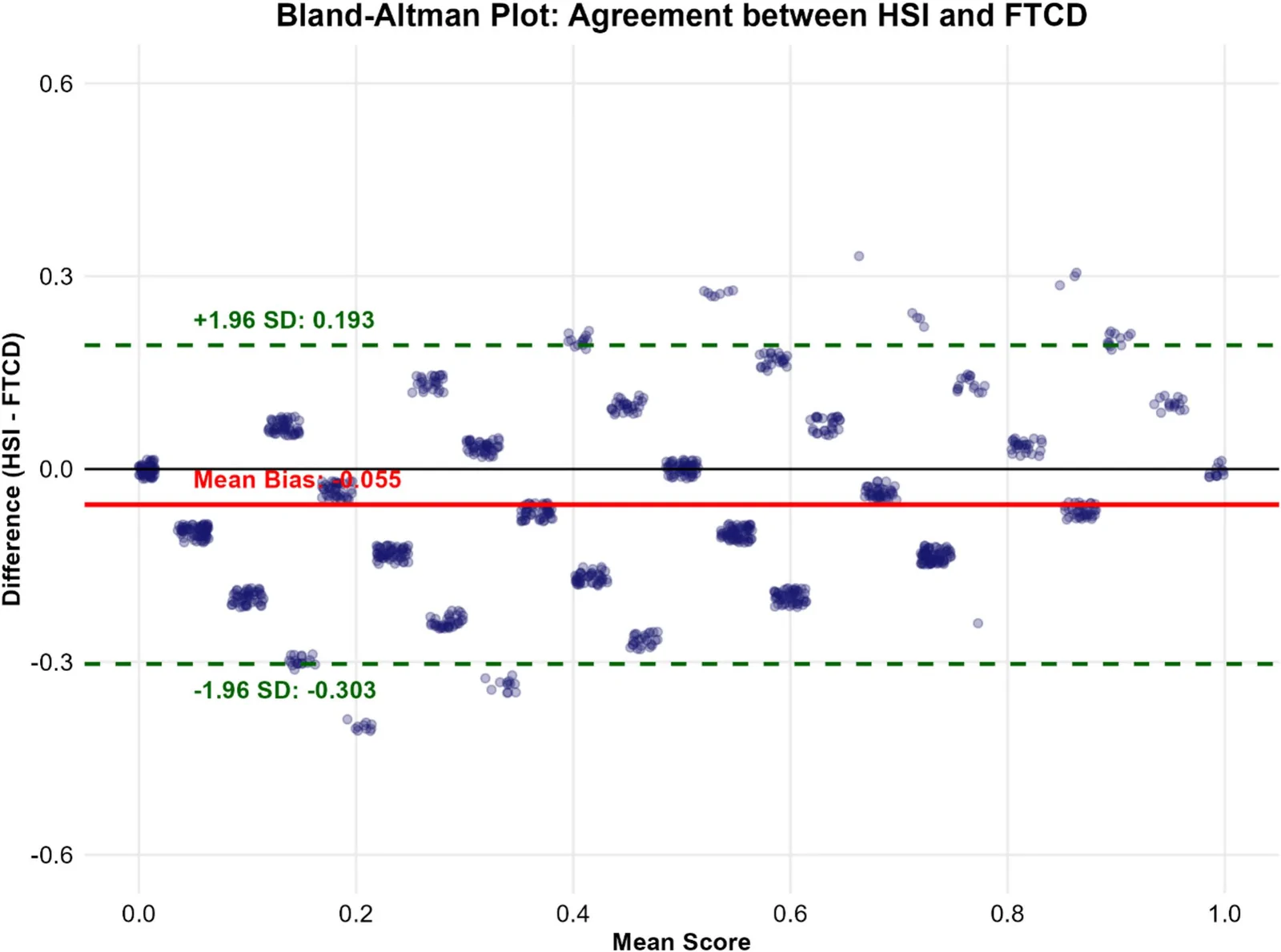
Bland–Altman plot for assessing systematic bias and agreement between the linearly rescaled (0–1) scores of the HSI and FTCD.

To evaluate agreement between the linearly rescaled (0–1) scores of the HSI and FTCD, a Bland–Altman plot was employed. The analysis revealed minimal mean bias (−0.055; 95% LoA: −0.28 to 0.17), with 92% of observations within the limits of agreement. However, proportional bias was evident: the HSI systematically underestimated addiction at higher severity levels (FTCD >7; mean difference = −0.12). These findings suggest caution when using the HSI for severe addiction cases (Fig. 1).

### 3.3 Apparent classification performance of the HSI (ROC analysis)

**Fig. 2:**
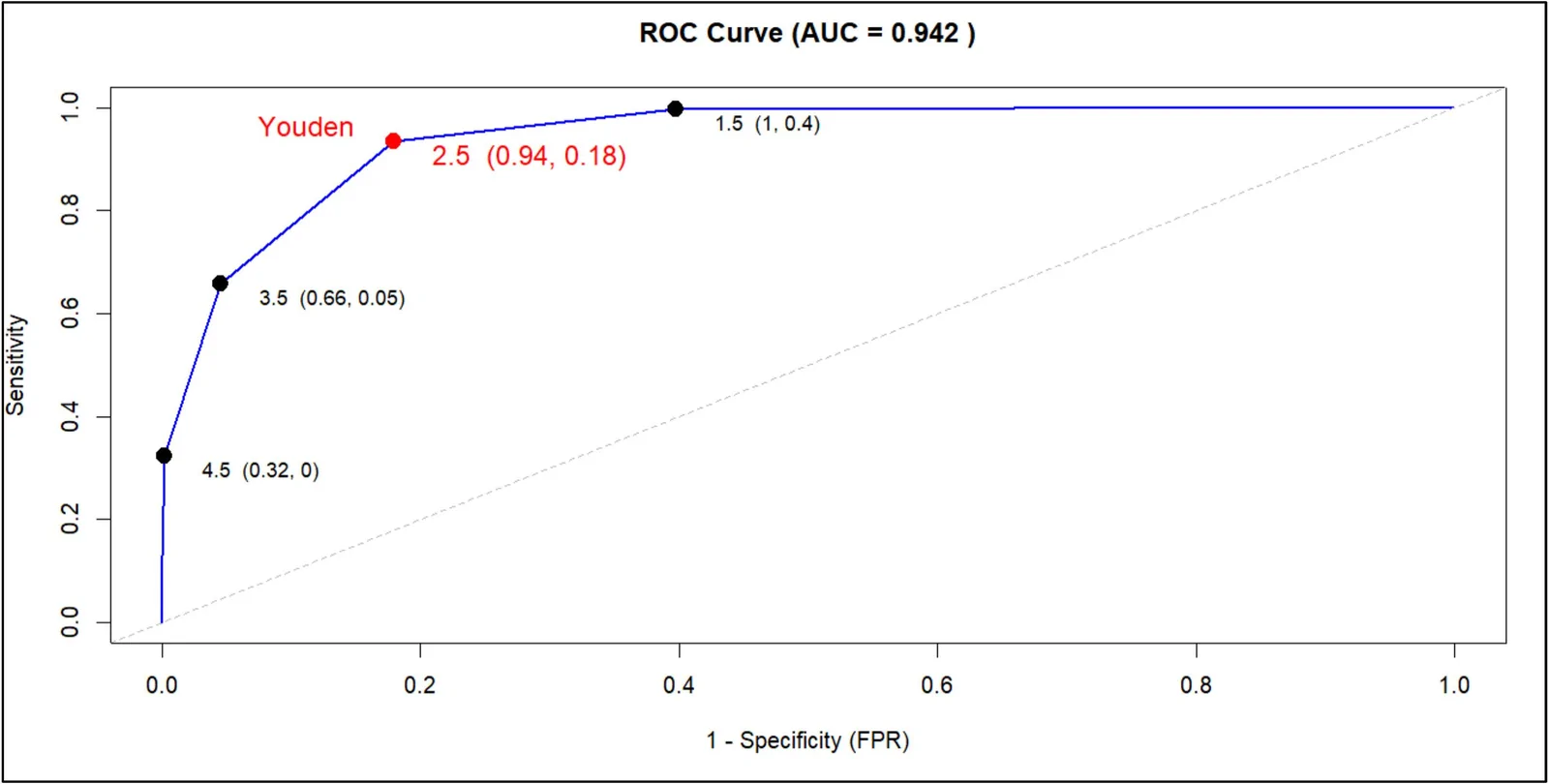
ROC curve illustrating the performance of the HSI in identifying individuals with high nicotine addiction. The optimal Youden cut-off (2.5) is rounded to ≥3 for clinical screening.

The ROC analysis yielded an AUC of 0.942. However, this value should be interpreted as a measure of apparent classification performance rather than true discriminative ability, since the HSI is derived from items within the FTCD (Fig. 2).

Analysis of different cutoff points showed that as the HSI score increases, sensitivity decreases while specificity increases. These findings should be interpreted with caution, as they reflect apparent classification performance due to the overlapping content between the HSI and FTCD. At a cutoff of 1.5, sensitivity was 99.8% with a specificity of 60.3%. At a cutoff of 2.5, sensitivity was 93.5% and specificity was 82.1%. At a cutoff of 3.5, sensitivity decreased to 65.9% while specificity increased to 95.5%. At a cutoff of 4.5, sensitivity was 32.4% with near-perfect specificity (99.9%).

The optimal cut-off point determined by the Youden index was 2.5. For screening purposes, a threshold of ≥3 was considered, while a threshold of ≥4 was examined for higher specificity. It is important to note that these cut-off values are based on apparent performance and have not been validated against an independent reference standard. Future studies comparing the HSI against external criteria such as DSM-5 tobacco use disorder diagnosis or biochemical markers are needed to determine the true clinical utility of these thresholds.

## 4 Subgroup analyses of agreement and convergent validity

### 4.1 Convergent validity across subgroups (pearson correlation)

**Fig. 3:**
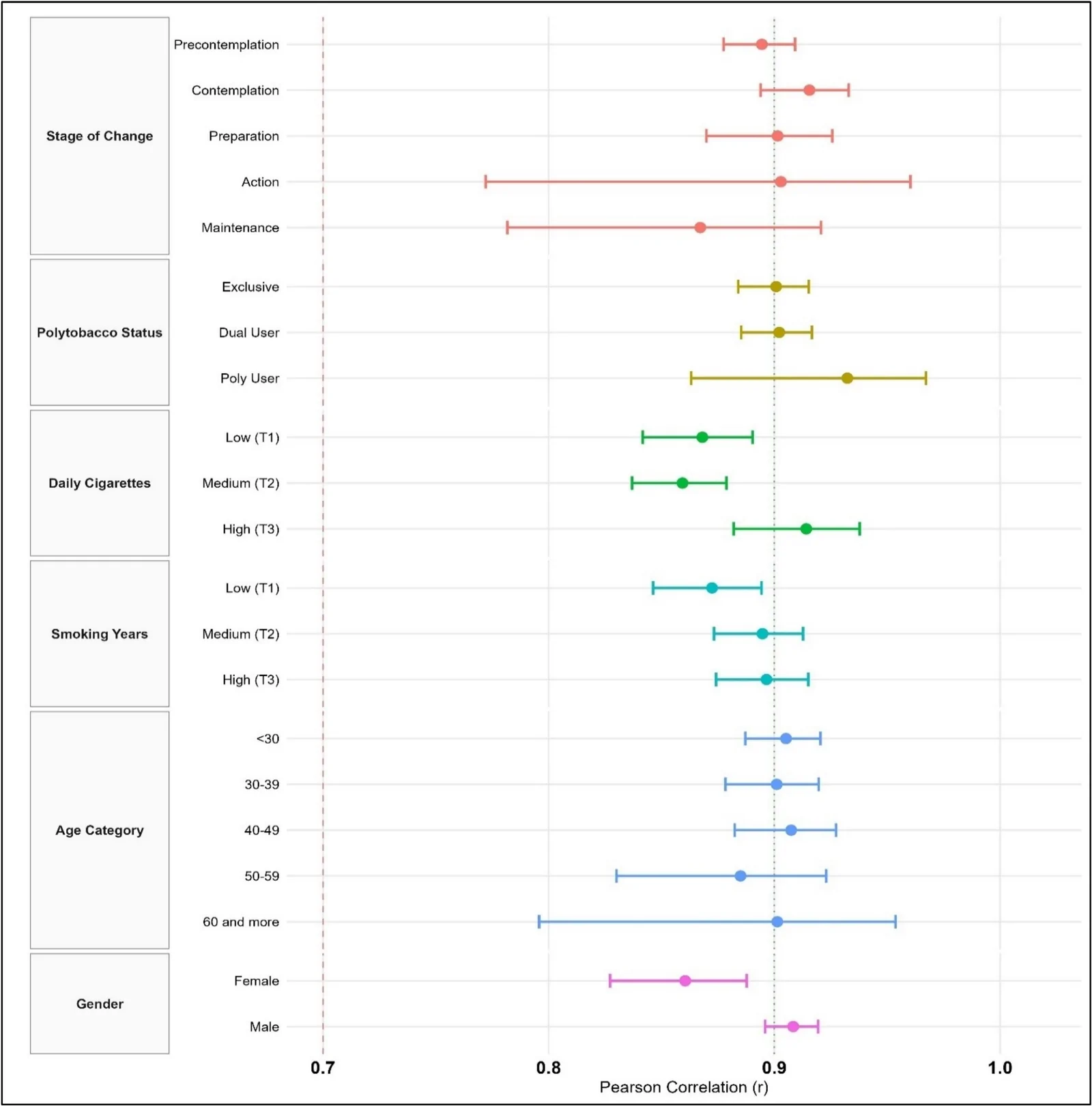
Linear correlation between FTCD and HSI scores across the study subgroups.

Pearson correlation analysis indicated a strong, positive, and direct relationship between the numerical scores of the FTCD questionnaire and the HSI across all subgroups. Correlation coefficients (r) ranged from 0.75 to 0.92 (*P* < 0.001). The highest correlation was observed in the poly-tobacco user subgroup, with *r* = 0.93. The narrow 95% confidence intervals in most groups reflect the high precision of the correlation estimates in this study (Fig. 3).

### 4.2 Continuous agreement across subgroups (ICC)

**Fig. 4:**
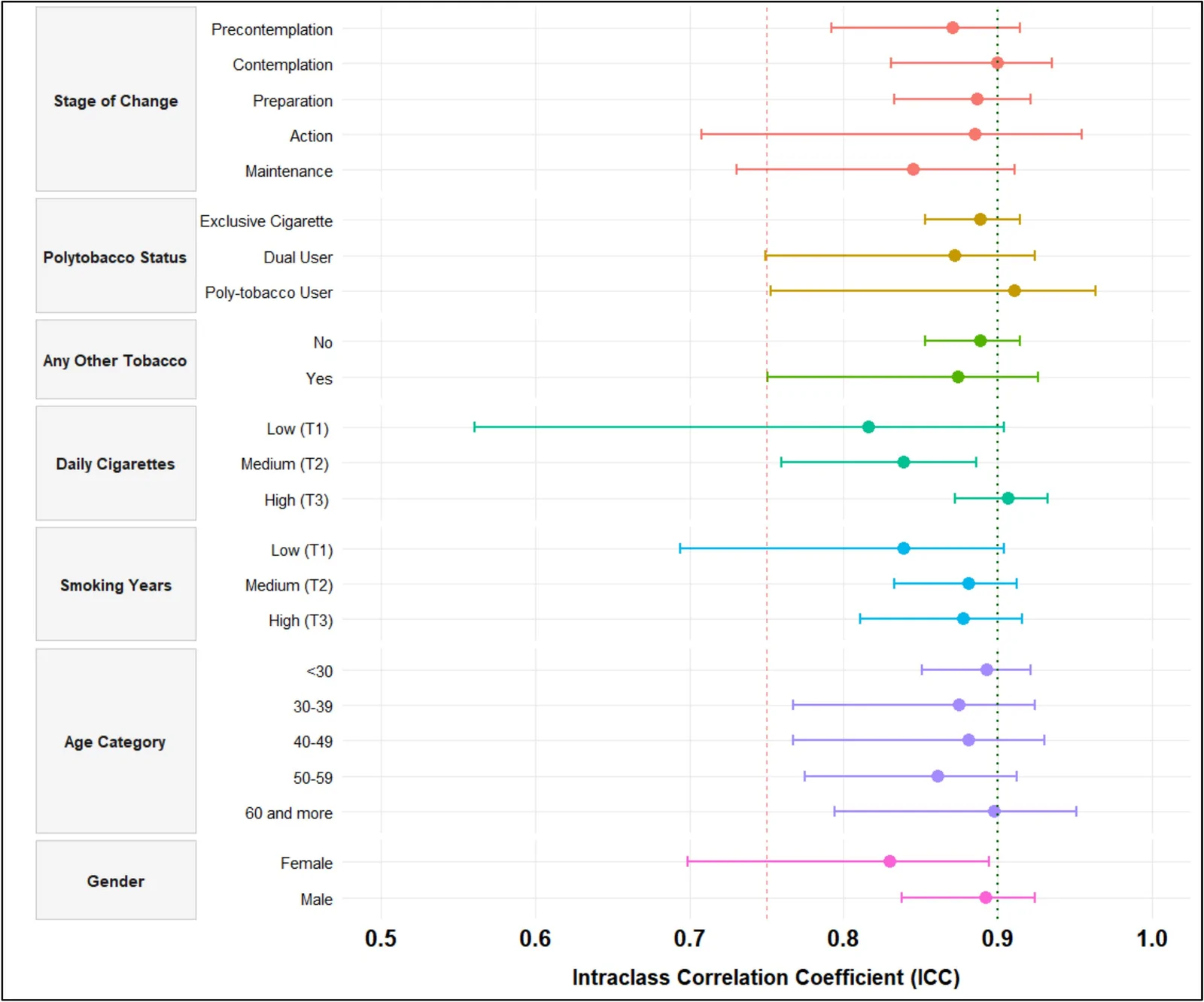
Assessment of reproducibility and reliability between FTCD and HSI across the study subgroups.

Subgroup analysis demonstrated that excellent agreement between the FTCD and HSI was maintained across all examined demographic and behavioral subgroups. ICC values in all 23 subgroups exceeded 0.84 (*P* < 0.001), indicating excellent agreement according to Cicchetti's criteria (Cicchetti, 1994). As shown in the Forest Plot, the highest agreement was observed among poly-tobacco users (ICC = 0.931), while the lowest was among moderate cigarette smokers (ICC = 0.845). The consistency of ICC estimates across demographic and behavioral strata suggests that the shortened HSI demonstrates agreement patterns broadly comparable to those of the FTCD across diverse smoking subgroups (Fig. 4).

### 4.3 Categorical agreement across subgroups (Kappa)

**Fig. 5:**
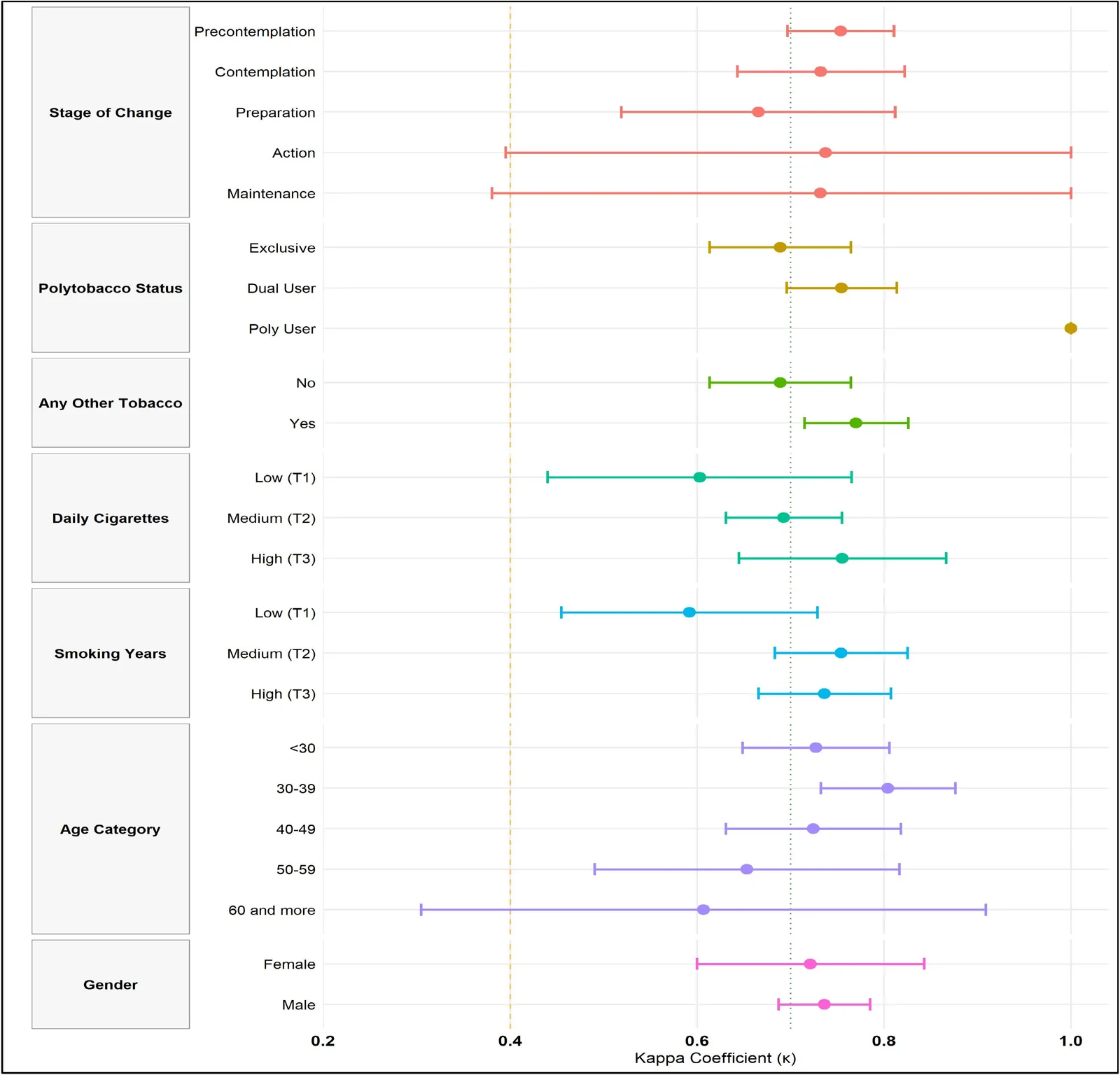
Results of subgroup agreement analysis using Cohen's Kappa (κ).

Analysis of agreement across different subgroups showed that Cohen's Kappa (κ) remained at a “moderate” to “substantial” level in all categories (κ range across subgroups: 0.42–0.78). Regarding behavioral variables, the highest agreement was observed in the high daily cigarette consumption group (κ ≈ 0.75), suggesting higher classification agreement at higher levels of cigarette consumption in identifying severe addiction. Furthermore, the stability of Kappa coefficients across all age and gender categories indicates that the performance of this instrument is not affected by demographic characteristics. In contrast, the lowest agreement was observed in the Action and Maintenance stages. The wider confidence intervals in these subgroups correspond to the limited sample sizes in these categories (Fig. 5).

### 4.4 Apparent classification performance across subgroups (ROC analysis)

Subgroup analyses were exploratory and intended to descriptively assess the consistency of HSI–FTCD agreement across different demographic and behavioral subgroups. Apparent classification agreement varied across subgroups, with AUC values ranging from 0.92 to 1.00. However, an AUC of 1.00 was observed only in very small subgroups (e.g., poly-tobacco users, *n* = 31; maintenance stage, *n* = 55), likely reflecting overfitting or statistical instability rather than true classification agreement. Consequently, these findings should be interpreted cautiously and not be overemphasized.

In contrast, among larger subgroups (*n* > 200), AUC values remained relatively consistent (0.92–0.96), suggesting stable apparent classification performance across the major demographic and behavioral strata. Consistent with the main analysis, the conventional HSI cut-off (≥4) generally provided higher specificity, whereas the Youden-derived cut-off (≥3) maximized screening sensitivity. Table 2 also reports the numbers of FTCD-positive and FTCD-negative participants and the 95% confidence intervals for AUC estimates to facilitate interpretation of subgroup-specific classification performance.

**Table 2:** Apparent classification performance of the heaviness of smoking index across demographic and behavioral subgroups.

Variable	Subgroup	N	FTCD+	FTCD−	AUC	Optimal cut-off			Standard cut-off (≥4)	
						Cut-off	Sensitivity	Specificity	Sensitivity	Specificity
Gender	Female	289	57	232	0.962 (0.943–0.981)	≥3	1.000	0.888	0.700	0.976
	Male	858	360	498	0.936 (0.922–0.950)	4	0.842	0.907	0.842	0.907
Age group (years)	<30	459	134	325	0.944 (0.926–0.962)	≥3	1.000	0.785	0.796	0.940
	30–39	324	129	195	0.955 (0.937–0.974)	4	0.870	0.940	0.870	0.940
	40–49	245	102	143	0.931 (0.903–0.958)	4	0.775	0.933	0.775	0.933
	50–59	91	40	51	0.926 (0.877–0.974)	4	0.885	0.828	0.885	0.828
	≥60	28	12	16	0.971 (0.926–1.000)	≥5	0.875	1.000	0.875	0.800
Smoking years	Low (T1)	383	65	318	0.948 (0.928–0.968)	≥3	1.000	0.862	0.600	0.965
	Medium (T2)	382	149	233	0.937 (0.916–0.957)	4	0.831	0.925	0.831	0.925
	High (T3)	382	203	179	0.932 (0.909–0.954)	4	0.879	0.868	0.879	0.868
Daily cigarette consumption	Low (T1)	383	50	333	0.971 (0.957–0.985)	≥3	1.000	0.892	0.552	0.984
	Medium (T2)	382	132	250	0.901 (0.874–0.929)	4	0.797	0.900	0.797	0.900
	High (T3)	382	235	147	0.922 (0.898–0.947)	≥5	0.916	1.000	0.976	0.754
Any other tobacco use	No	623	274	349	0.943 (0.928–0.958)	≥3	1.000	0.773	0.808	0.922
	Yes	524	143	381	0.948 (0.932–0.964)	4	0.831	0.932	0.831	0.932
Poly-tobacco status	Exclusive cigarette user	567	155	412	0.947 (0.931–0.963)	≥3	1.000	0.773	0.808	0.922
	Dual user	549	246	303	0.939 (0.923–0.956)	4	0.818	0.929	0.818	0.929
	Poly-tobacco user	31	16	15	0.975 (0.931–1.000)	4	1.000	1.000	1.000	1.000
Stage of change	Precontemplation	620	262	358	0.940 (0.924–0.956)	4	0.810	0.935	0.810	0.935
	Contemplation	271	102	169	0.953 (0.933–0.974)	4	0.853	0.903	0.853	0.903
	Preparation	180	43	137	0.936 (0.905–0.967)	4	0.815	0.922	0.815	0.922
	Action	21	6	15	0.950 (0.866–1.000)	≥3	1.000	0.812	0.800	0.938
	Maintenance	55	4	51	0.993 (0.977–1.000)	4	1.000	0.962	1.000	0.962

Abbreviations: AUC, area under the receiver operating characteristic curve; CI, confidence interval; FTCD+, participants with severe nicotine addiction according to the FTCD; FTCD−, participants without severe nicotine addiction according to the FTCD.

“Standard cut-off (≥4)” indicates the conventional HSI threshold for identifying severe nicotine addiction. “Optimal cut-off” indicates the threshold determined using Youden's J statistic. The Youden-derived cut-off of 2.5 is presented as ≥3 for practical screening purposes.

AUC estimates from small subgroups should be interpreted cautiously because limited sample sizes may result in wider confidence intervals and less stable estimates.

## 5 Discussion

Assessing nicotine addiction in cigarette smokers is essential for both clinical practice and epidemiological research, particularly for informing smoking cessation interventions. The present study evaluated the correlation, agreement, apparent classification performance, and subgroup stability of the HSI in comparison with the Fagerström Test for Cigarette Dependence (FTCD), with particular emphasis on the stability of these measures across key demographic and behavioral subgroups.

The very high correlation and excellent agreement observed between the HSI and FTCD indicate that the two instruments rank nicotine addiction severity in a highly consistent manner. The intraclass correlation coefficient demonstrated excellent agreement (ICC = 0.950), reflecting strong reproducibility of HSI scores relative to FTCD. At the categorical level, Cohen's Kappa indicated substantial agreement (κ = 0.645), suggesting that although the instruments are largely concordant, some degree of individual-level misclassification may occur due to differences in scale structure and scoring approaches. Overall, these findings support the conceptual overlap between the HSI and FTCD while acknowledging that the FTCD captures additional dimensions of addiction beyond consumption intensity. The HSI's brevity (2 versus 6 items) offers practical advantages by reducing respondent burden and administration time, making it suitable for rapid screening in busy clinical settings and large epidemiological surveys (Borland et al., 2010; Heatherton et al., 1991). The instrument demonstrated excellent reliability in this study (ICC = 0.950).

These results are broadly consistent with previous population-based studies reporting strong correlations and substantial agreement between the HSI and FTCD. Pérez-Ríos et al., for example, demonstrated high concordance between the two instruments in large samples from Spain and the United States, with minor variations across demographic subgroups such as gender and smoking intensity (Pérez-Ríos et al., 2009). Similarly, a previous Iranian study reported substantial agreement between the HSI and FTCD in a sample of male smokers (Pahlavanzadeh & Charkazi, 2024). While that study and the present findings demonstrate good agreement between the two questionnaires in Iranian smokers, it should be noted that existing Iranian studies have primarily focused on the psychometric properties (such as reliability and factor structure) of the Persian versions rather than criterion validity against independent clinical or biological standards.

In contrast, some clinical studies have reported more moderate agreement between the HSI and FTCD, particularly at higher levels of addiction, suggesting potential divergence between the two measures in heavily addicted smokers (Lim et al., 2022). Although the mean bias observed in our Bland–Altman analysis was minimal, evidence of proportional bias was present, with the HSI tending to underestimate addiction severity among participants with higher FTCD scores (>7). These findings are broadly consistent with population-based studies reporting strong overall agreement between the HSI and FTCD (Chabrol et al., 2005; Pérez-Ríos et al., 2009), while also suggesting that caution may be warranted when interpreting HSI scores among heavily addicted smokers. Variations across studies may stem from differences in study setting (e.g., clinical versus community samples), sample characteristics (e.g., age, gender, and poly-tobacco use), and cultural or behavioral smoking patterns. For instance, in Iran, the concurrent use of waterpipe (hookah) and cigarettes may influence smoking patterns, including time to first cigarette and daily cigarette consumption, potentially affecting HSI performance. Similarly, social smoking practices and cultural norms may influence responses to FTCD items and contribute to differences in agreement estimates across populations.

Overall, these findings indicate a high level of concordance between the HSI and the FTCD, while acknowledging the substantial overlap between the two instruments. Within this context, the HSI may serve as a practical brief screening tool when rapid assessment is required. However, this conclusion should be interpreted in light of the shared item content between the two instruments and does not constitute evidence of independent criterion validity against clinical or biological measures in Iranian populations.

Subgroup analyses showed that the correlation and agreement between the HSI and FTCD were generally stable across most demographic variables, reinforcing the generalizability of the HSI across heterogeneous smoking populations. Although minor variations were observed across some subgroups, overall concordance remained consistent across gender and age categories.

These observations are broadly consistent with previous studies suggesting that gender has little influence on the performance of the FTCD or its agreement with the HSI. While some studies have reported modest gender-related differences, others found no significant effect of gender on FTCD scores (Chabrol et al., 2005; Chinwong et al., 2018; Lim et al., 2022; Põld & Pärna, 2020).

Older age was associated with higher sensitivity of the HSI. This association may be explained by the possibility that older smokers have more established smoking routines and more stable cigarette consumption patterns; however, this interpretation is speculative and requires confirmation in future studies. These results suggest that certain demographic characteristics may influence the accuracy of brief consumption-based indices in detecting severe nicotine addiction.

Regarding behavioral characteristics, higher correlation coefficients, higher ICC and Cohen's kappa values, and greater sensitivity at the standard HSI cut-off were generally observed among participants with greater cigarette consumption, longer smoking histories, and more complex tobacco use behaviors. These subgroup patterns indicate variation in the magnitude of concordance between the HSI and FTCD across behavioral strata while remaining consistently high overall. Importantly, categorical agreement remained acceptable among lighter and exclusive cigarette smokers, supporting the applicability of the HSI across varying levels of tobacco use. Similar findings have been reported in large samples of smokers, where the HSI and FTCD showed comparable performance in identifying severe nicotine addiction, particularly among heavier smokers (Chabrol et al., 2005).

Subgroup-specific ROC estimates should be interpreted with caution, particularly for subgroups with limited sample sizes or few FTCD-positive and FTCD-negative participants. Although some small subgroups demonstrated excellent diagnostic performance, including an AUC of 1.00 among poly-tobacco users, these estimates may be less stable and should not be interpreted as evidence of superior discrimination. The inclusion of FTCD-positive and FTCD-negative counts in Table 2 allows readers to assess the robustness of subgroup-specific ROC estimates.

ROC analysis demonstrated very high apparent classification performance of the HSI (AUC = 0.942) for identifying severe nicotine addiction based on FTCD classification. Evaluation of different cut-off points indicated that a score of 2.5 (Youden index) provided an optimal balance between sensitivity and specificity for screening high nicotine addiction. For clinical application, this threshold can be rounded to ≥3, which maintains good sensitivity (89%) and specificity (85%). A higher cut-off of ≥4 is recommended when high specificity is required for rigorous identification of severe addiction with minimized false-positive rates. Higher cut-off points increased specificity at the expense of sensitivity, particularly among lighter smokers or individuals in earlier stages of behavior change. These findings are consistent with previous research emphasizing the need to adjust HSI cut-off points based on study objectives and population characteristics(Etter et al., 2003; Fagerström, 2012).

At a cut-off point of 4, specificity remained consistently high across subgroups, whereas sensitivity varied by addiction e severity and behavioral stage. Sensitivity was higher among men, older individuals, heavier smokers, and those in more advanced stages of behavior change, indicating that higher thresholds may be more suitable for identifying individuals with definite high severity rather than for initial screening purposes.

The Bland–Altman analysis showed a minimal mean bias, with approximately 92% of observations falling within the limits of agreement. These results should be interpreted in the context of the structural relationship between the two instruments, as the HSI is a short-form measure derived from two items of the FTCD. Therefore, the observed agreement reflects comparison between a brief instrument and its parent scale rather than between two independent measures and should not be interpreted as evidence of independent criterion validity. Consistent with the proportional bias observed, the HSI systematically underestimated addiction severity at higher FTCD scores (>7). While previous studies have reported acceptable agreement between the HSI and FTCD (Chabrol et al., 2005; Pérez-Ríos et al., 2009), these findings should be interpreted cautiously due to overlapping item content. Accordingly, potential underestimation at higher severity levels should be considered when applying the HSI in clinical settings.

The findings of the present study largely supported the stated hypotheses. Consistent with the first hypothesis, the HSI demonstrated a strong positive correlation and excellent agreement with the FTCD, as evidenced by the high Pearson correlation coefficient and intraclass correlation coefficient. The second hypothesis was also supported, with Cohen's kappa indicating substantial agreement between the two instruments in classifying smokers according to levels of nicotine addiction. In line with the third hypothesis, the HSI showed good discriminatory ability in identifying high nicotine addiction, as reflected by the high area under the ROC curve. Finally, although some minor variations were observed, the overall concordance between the HSI and FTCD, as well as the apparent classification performance of the HSI, remained relatively stable across most demographic and behavioral subgroups, providing partial support for the fourth hypothesis.

Taken together, these results indicate that the HSI shows good agreement with the FTCD and may serve as a practical brief screening tool when rapid assessment is needed. However, given that existing evidence in Iranian populations is largely limited to psychometric properties and questionnaire agreement rather than criterion validity, caution is warranted when interpreting HSI scores as a measure of nicotine addiction severity.

Furthermore, while the present study focused on the agreement between HSI and FTCD, it is important to consider the predictive utility of HSI in relation to real-world cessation behaviors. Jena et al. (2024), using data from eight African countries, examined the association between HSI scores and quit attempts as well as quit intentions among daily cigarette smokers. Their findings indicated only limited associations between low nicotine addiction (HSI < 2) and quit-related behaviors, suggesting that the relationship between addiction measures and cessation outcomes may be context-dependent and influenced by cultural and usage patterns. These results underscore that strong agreement between HSI and FTCD does not necessarily imply strong predictive validity for cessation outcomes. Future research in the Iranian context could therefore benefit from evaluating the HSI against behavioral endpoints such as quit attempts and sustained abstinence to better establish its practical utility.

### 5.1 Strengths of the study

This study had several key strengths. First, in addition to a large and diverse sample size, the inclusion of female smokers improved the representativeness of the sample and allowed for the evaluation of the tools' performance across both genders, which was limited to males in a previous study in Iran (Pahlavanzadeh & Charkazi, 2024). Second, the use of multiple complementary agreement metrics rather than reliance on correlation alone—comprising Pearson correlation, ICC, Cohen's Kappa, ROC, and Bland–Altman analyses—enabled a comprehensive evaluation of agreement and classification agreement. Furthermore, subgroup analyses and the identification of optimal cut-off points enhanced the practical relevance of the findings for both clinical and research applications.

### 5.2 Study limitations

This study had several limitations that should be considered when interpreting the results. First, the cross-sectional design precludes causal inference or evaluation of changes in cigarette addiction over time. Second, reliance on self-reported data may have introduced recall and social desirability bias. Third, the use of convenience sampling, which was chosen primarily for feasibility reasons, may limit the generalizability of the findings to the broader population of Iranian smokers.

Fourth, HSI is derived from two FTCD items, creating criterion contamination and overlap that likely inflated agreement and ROC metrics (e.g., r, ICC, and AUC). Thus, the findings primarily reflect internal consistency and apparent performance rather than true independent classification agreement validity. Future studies should evaluate the performance of HSI against independent criteria such as biomarkers (e.g., expired CO or urinary cotinine), multidimensional addiction scales, or clinically relevant outcomes.

Fifth, inclusion criteria (e.g., ≥10 cigarettes/week and ≥ 100 lifetime cigarettes) may have included non-daily smokers, although FTCD and HSI are primarily optimized for daily smoking patterns. Sixth, subgroup analyses were exploratory and descriptive in nature, and no formal statistical comparisons were conducted between subgroups. Therefore, no multiplicity adjustment was applied, and subgroup findings—particularly those from smaller samples—should be interpreted cautiously due to possible statistical instability and imprecise estimates.

Seventh, the present study did not include an independent psychometric evaluation of the HSI (e.g., factor analysis or test–retest reliability). Furthermore, the relatively small number of participants in the Action (*n* = 21) and maintenance (*n* = 55) stages may have reduced the stability of subgroup-specific estimates. Eighth, FTCD and HSI were originally developed and validated for exclusive cigarette smokers. However, nearly half of our sample consisted of dual or poly-tobacco users (e.g., concurrent use of hookah, nass, or vaping). Because these instruments do not capture addiction on non-cigarette nicotine products, the total severity of nicotine addiction in mixed-product users may have been underestimated. This limitation represents a potential threat to the construct validity of the study findings. Although conducting a sensitivity analysis restricted to exclusive cigarette smokers would have been valuable, the available data did not allow reliable identification of exclusive users. Therefore, caution is warranted when generalizing the results to populations with high rates of poly-tobacco use.

Furthermore, the inclusion of participants in the action and maintenance stages of smoking cessation may have introduced recall bias and potential misclassification. Although these participants were instructed to respond to the FTCD and HSI items based on their past smoking behavior, retrospective assessment of nicotine addiction is inherently subject to memory distortion and may not accurately reflect their addiction level at the time they were actively smoking. This could have affected the agreement estimates between HSI and FTCD, particularly in subgroup analyses. Future studies should preferably restrict analyses to individuals who are currently smoking to minimize such biases.

### 5.3 Implications

These findings have several implications. Research-wise, future studies should validate HSI against independent tools (e.g., QSU) in diverse Iranian subpopulations—including poly-tobacco users (dual: 47.9%; poly: 2.7%)—where FTCD /HSI applicability is limited by underestimating non-cigarette addiction (hookah/nass/vaping). Multi-product addiction measures are needed. Clinically, HSI's brevity supports its use in busy primary care for quick screening, reserving FTCD for detailed assessments. For public health, high HSI/FTCD agreement enables efficient addiction monitoring in community programs, informing targeted interventions like brief counseling for light smokers (80.8% of our sample) prevalent in Iran, potentially enhancing quitline efficacy and policy evaluations.

## 6 Conclusion

The HSI is a brief and practical instrument for the rapid assessment of cigarette addiction. The present findings demonstrated a high level of concordance with the FTCD, reflected by strong correlation and excellent agreement between the two instruments. These findings support the use of the HSI as a brief screening instrument when rapid assessment is required, rather than as a replacement for the FTCD. In particular, the HSI may be useful for identifying individuals classified by the FTCD as having severe cigarette addiction. Nevertheless, this interpretation should be considered in light of the fact that the apparent classification performance was evaluated against its parent instrument rather than an independent reference standard. However, the HSI tended to underestimate addiction severity at higher FTCD scores, indicating that the FTCD may remain preferable when a more comprehensive assessment is required. For screening purposes, an HSI cut-off of ≥3 (rounded from the optimal Youden cut-off of 2.5) is recommended, whereas a cut-off of ≥4 provides higher specificity for identifying severe addiction. Because the HSI is derived from FTCD items, these findings reflect agreement and apparent classification performance rather than independent criterion validity. Future studies should validate the HSI against external clinical, behavioral, or biological criteria in diverse populations.

## List of Abbreviations

**Table:** 

FTCD	Fagerström Test for Cigarette Dependence
HSI	Heaviness of Smoking Index
ROC	Receiver Operating Characteristic
ICC	Intraclass Correlation Coefficient
WISDM	Wisconsin Inventory of Smoking Dependence Motives
NDSS	Nicotine Dependence Syndrome Scale
QSU	Questionnaire of Smoking Urges
FTND	Fagerström Test for Nicotine Dependence

## Declaration of AI use

The authors declare that artificial intelligence tools were used solely for language editing and improving clarity. The content, analysis, and interpretation of data were entirely conducted by the authors, who take full responsibility for the integrity of the work.

## CRediT authorship contribution statement

**Abdurrahman Charkazi:** Writing – review & editing, Writing – original draft, Validation, Supervision, Resources, Methodology, Investigation, Formal analysis, Data curation, Conceptualization. **Seyed Ghadir Hosseini:** Writing – review & editing, Resources, Investigation, Conceptualization. **Hajar Dehghan:** Writing – review & editing, Resources, Investigation, Conceptualization. **Arezou Foroughi:** Writing – review & editing, Resources, Investigation, Conceptualization. **Bagher Pahlavanzadeh:** Writing – review & editing, Visualization, Software, Methodology, Formal analysis.

## Consent to participate

Informed consent was obtained from all participants prior to the interview. Participants were assured that their responses would be kept confidential.

## Ethical approval

The Institutional Review Board of Golestan University of Medical Sciences, Vice-Chancellery for Research and Technology approved the study protocol (IR.GOUMS.REC.1404 .084).

## Funding

Golestan University of Medical Sciences (Grant No. 114248).

## Declaration of competing interest

The authors declare the following financial interests/personal relationships which may be considered as potential competing interests: [The authors, Abdurrahman Charkazi, Seyed Ghadir Hosseini, Hajar Dehghan, Arezou Foroughi, and Bagher Pahlavanzadeh, declare that they have no known competing financial interests or personal relationships that could have appeared to influence the work reported in this paper. All authors have read and approved the content of this declaration].

## Data Availability

Data will be made available on request.

## References

[bb0005] Abbasi-Kangevari M., Ghanbari A., Fattahi N., Malekpour M.R., Masinaei M., Ahmadi N., Farzadfar F. (2023). Tobacco consumption patterns among Iranian adults: A national and sub-national update from the STEPS survey 2021. Scientific Reports.

[bb0010] American Psychiatric Association (2022).

[bb0015] Benowitz N.L., Henningfield J.E. (1994). Establishing a nicotine threshold for addiction. The implications for tobacco regulation. The New England Journal of Medicine.

[bb0020] Benowitz N.L., Porchet H., Sheiner L., Jacob P. (1988). Nicotine absorption and cardiovascular effects with smokeless tobacco use: Comparison with cigarettes and nicotine gum. Clinical Pharmacology and Therapeutics.

[bb0025] Borland R., Yong H.H., O’Connor R.J., Hyland A., Thompson M.E. (2010). The reliability and predictive validity of the heaviness of smoking index and its two components: Findings from the international Tobacco control four country study. Nicotine & Tobacco Research.

[bb0030] Centers for Disease Control and Prevention (CDC) (2023). Behavioral Risk Factor Surveillance System Survey Data. https://www.cdc.gov/brfss/.

[bb0035] Chabrol H., Niezborala M., Chastan E., de Leon J. (2005). Comparison of the heavy smoking index and of the Fagerstrom test for nicotine dependence in a sample of 749 cigarette smokers. Addictive Behaviors.

[bb0040] Chaiton M.O., Cohen J.E., McDonald P.W., Bondy S.J. (2007). The heaviness of smoking index as a predictor of smoking cessation in Canada. Addictive Behaviors.

[bb0045] Chinwong D., Mookmanee N., Chongpornchai J., Chinwong S. (2018). A comparison of gender differences in smoking behaviors, intention to quit, and nicotine dependence among Thai university students. Journal of Addiction.

[bb0050] Cicchetti D.V. (1994). Guidelines, criteria, and rules of thumb for evaluating normed and standardized assessment instruments in psychology. Psychological Assessment.

[bb0055] Darville A., Hahn E.J. (2014). Hardcore smokers: What do we know?. Addictive Behaviors.

[bb0060] Diaz F.J., Jané M., Saltó E., Pardell H., Salleras L., Pinet C., de Leon J. (2005). A brief measure of high nicotine dependence for busy clinicians and large epidemiological surveys. Australian and New Zealand Journal of Psychiatry.

[bb0065] Etter J.-F. (2005). A comparison of the content-, construct-and predictive validity of the cigarette dependence scale and the Fagerström test for nicotine dependence. Drug and Alcohol Dependence.

[bb0070] Etter J.-F., Le Houezec J., Perneger T.V. (2003). A self-administered questionnaire to measure dependence on cigarettes: The cigarette dependence scale. Neuropsychopharmacology.

[bb0075] Fagerström K. (2012). Determinants of tobacco use and renaming the FTND to the Fagerström test for cigarette dependence. Nicotine & Tobacco Research.

[bb0080] Fiore M.C., Baker T.B. (2011). Clinical practice. Treating smokers in the health care setting. New England Journal of Medicine.

[bb0085] Fiore M.C., Jaén C.R., Baker T.B. (2008). https://stacks.cdc.gov/view/cdc/6964/cdc_6964_DS1.pdf.

[bb0090] Flor L.S., Reitsma M.B., Gupta V., Ng M., Gakidou E. (2021). The effects of tobacco control policies on global smoking prevalence. Nature Medicine.

[bb0095] GBD 2019 Tobacco Collaborators. (2021). Spatial, temporal, and demographic patterns in prevalence of smoking tobacco use and attributable disease burden in 204 countries and territories, 1990-2019: A systematic analysis from the global burden of disease study 2019. The Lancet.

[bb0100] Glover E.D., Nilsson F., Westin Å., Glover P.N., Laflin M.T., Persson B. (2005). Developmental history of the Glover-Nilsson smoking behavioral questionnaire. American Journal of Health Behavior.

[bb0105] Heatherton T.F., Kozlowski L.T., Frecker R.C., Fagerström K.O. (1991). The Fagerström test for nicotine dependence: A revision of the Fagerstrom tolerance questionnaire. British Journal of Addiction.

[bb0110] Heatherton T.F., Kozlowski L.T., Frecker R.C., Rickert W., Robinson J. (1989). Measuring the heaviness of smoking: Using self-reported time to the first cigarette of the day and number of cigarettes smoked per day. British Journal of Addiction.

[bb0115] Jena P.K., Okoye P.C., Patel A., Satpathy N., Kamsa-ard S., Mohapatra D., Justin E.K. (2024). Association between the heaviness of smoking index (HSI) and quit attempts and quit intentions among cigarette smokers in eight African countries. Discover Public Health.

[bb0120] Kozlowski L.T., Porter C.Q., Orleans C.T., Pope M.A., Heatherton T. (1994). Predicting smoking cessation with self-reported measures of nicotine dependence: FTQ, FTND, and HSI. Drug and Alcohol Dependence.

[bb0125] Lim K.H., Cheong Y.L., Sulaiman N., Yah X.Y., Mahadzir M.E., Lim J.H., Lim H.L. (2022). Agreement between the Fagerström test for nicotine dependence (FTND) and the heaviness of smoking index (HSI) for assessing the intensity of nicotine dependence among daily smokers. Tobacco Induced Diseases.

[bb0130] National Cancer Institute (2010). https://progressreport.cancer.gov/.

[bb0135] Pahlavanzadeh B., Charkazi A. (2024). The agreement between the Fagerström test for nicotine dependence and the heaviness of smoking index among Iranian male smokers. Addiction & Health.

[bb0140] Pérez-Ríos M., Santiago-Pérez M.I., Alonso B., Malvar A., Hervada X., de Leon J. (2009). Fagerstrom test for nicotine dependence vs heavy smoking index in a general population survey. BMC Public Health.

[bb0145] Piper M.E., Piasecki T.M., Federman E.B., Bolt D.M., Smith S.S., Fiore M.C., Baker T.B. (2004). A multiple motives approach to tobacco dependence: The Wisconsin inventory of smoking dependence motives (WISDM-68). Journal of Consulting and Clinical Psychology.

[bb0150] Põld M., Pärna K. (2020). Nicotine dependence and factors related to smoking cessation among physicians in Estonia. International Journal of Environmental Research and Public Health.

[bb0155] Prochaska J.O., DiClemente C.C. (1983). Stages and processes of self-change of smoking: Toward an integrative model of change. Journal of Consulting and Clinical Psychology.

[bb0160] Rolstad S., Adler J., Rydén A. (2011). Response burden and questionnaire length: Is shorter better? A review and meta-analysis. Value in Health.

[bb0165] Sarbandi F., Niknami S., Hidarnia A., Hajizadeh E., Azaripour M.H., Eslampanah N.S. (2015). Psychometric properties of the Iranian version of the fagerstrom test for nicotine dependence and of heaviness of smoking index. Journal of Research and Health.

[bb0170] Shiffman S., Waters A., Hickcox M. (2004). The nicotine dependence syndrome scale: A multidimensional measure of nicotine dependence. Nicotine & Tobacco Research.

[bb0175] U.S. Department of Health and Human Services (1989). Reducing the health consequences of smoking*: 25 years of progress* (DHHS Publication No. CDC 89–8411). https://profiles.nlm.nih.gov/101584932X393.

[bb0180] U.S. Department of Health and Human Services (2020). https://www.cdc.gov/tobacco/data_statistics/sgr/2020-smoking-cessation/index.html.

[bb0185] Wellman R.J., DiFranza J.R., Savageau J.A., Godiwala S., Friedman K., Hazelton J. (2005). Measuring adults’ loss of autonomy over nicotine use: The hooked on nicotine checklist. Nicotine & Tobacco Research.

[bb0190] World Health Organization (2019). Strengthening health systems for treating tobacco dependence in primary care. https://www.who.int/publications/i/item/strengthening-health-systems-for-treating-tobacco-dependence-in-primary-care.

